# Forward and back is not enough: applying best practices for translation of pediatric sleep questionnaires

**DOI:** 10.3389/frsle.2023.1329405

**Published:** 2024-01-16

**Authors:** Darcy A. Thompson, Melissa S. Fineman, Estefania Miramontes Valdes, Jeanne M. Tschann, Lisa J. Meltzer

**Affiliations:** ^1^Department of Pediatrics, University of Colorado School of Medicine, Aurora, CO, United States; ^2^Adult and Child Center for Outcomes Research and Delivery Science, University of Colorado School of Medicine, Aurora, CO, United States; ^3^Department of Psychiatry and Behavioral Sciences, University of California, San Francisco, San Francisco, CA, United States; ^4^Department of Pediatrics, National Jewish Health, Denver, CO, United States; ^5^Department of Family Medicine, University of Colorado School of Medicine, Aurora, CO, United States

**Keywords:** cultural adaptation, culture, Spanish, qualitative, survey, pretesting, decentering, cognitive interview

## Abstract

Cultural differences in the experience of sleep warrant consideration in the measurement of sleep across populations. This requires careful attention to both language and culture when translating survey measures. While forward and back translation is the most commonly used approach, it has numerous limitations if used as an isolated method. Best practice guidelines recommend a multi-step team-based approach for translating questionnaires. We present our recent experience applying best practices in a study with both Spanish and English-speaking Mexican American mothers of toddlers. This work is part of a larger project that will measure parental sleep-related beliefs and parenting practices in Mexican American parents of toddlers. We utilized a team-based approach to translation and cultural adaptation, assembling a diverse, bilingual, and bicultural team. The translation process started with items and measures that we had selected, revised as needed, or created. New items were based on constructs identified in semi-structured interviews and focus groups used to explore parental sleep-related beliefs and parenting practices in the target population. Following this, our translation process included forward and back translation, harmonization and decentering, cognitive interviewing, debriefing, adjudication, and proofreading. We outline details of our process and the rationale for each step. We also highlight how each step contributes to ensuring culturally appropriate items with conceptual equivalence across languages. To ensure inclusivity and scientific rigor within the field of sleep research, investigators must utilize best practices for translations and cultural adaptations, building on the foundation of cultural constructs often identified in qualitative work.

## 1 Introduction

The subjective experience of sleep can only be captured through patient-reported outcomes (PROs). Unlike polysomnography or actigraphy which objectively assess sleep-wake patterns, beliefs about sleep, sleep quality, and sleep-related behaviors must be reported by the patient. In pediatrics, the patient may be too young to report on the subjective experience, thus we often rely on parent-report measures, which may include sleep-related parenting practices, parental beliefs about sleep, and sleep location. There are over 240 pediatric sleep questionnaires that aim to capture the subjective experience of sleep. However, only about 7% of these tools are available in more than one language (Spruyt and Gozal, 2011; Sen and Spruyt, 2020).

While sleep is a universal experience, there are linguistic and cultural differences in sleep practices, sleep schedules, and beliefs about sleep behavior around the world. For example, a recent study considered the limitations of English-language concepts around sleep in families, reporting a Czech word—Uspávání—which describes calming parenting practices to help young children fall asleep and stay asleep (Tinková and Ball, 2022). Similar words are found in several other languages, all referring to parental assistance or parental presence when a child is falling asleep (Tinková and Ball, 2022). This highlights the need for careful attention to language and culture in sleep-related questionnaires, utilizing appropriate methods for both translation and cultural adaptation.

Over time, the approach to questionnaire translation has evolved from simple forward translation to much more complex approaches including both linguistic translations and cultural adaptations. This is currently reflected within the field of sleep research with investigators most commonly applying simple approaches, like forward and back translation. Beyond putting a higher value on the original language, known weaknesses of forward and back translation include word-for-word translation and a loss of the original conceptual meaning (Harkness, 2003; Cheng and Im, 2021).

Best practice guidelines recommend a multi-step team-based approach for translating questionnaires from one language to another including a combination of forward translation, back translation, reconciliation, harmonization, decentering, pretesting (e.g, cognitive interviewing), cognitive debriefing, adjudication, and proofreading (Wild et al., 2005, 2009; Acquadro et al., 2008; Pennell and Cibelli Hibben, 2016; Eremenco et al., 2018; Walde and Völlm, 2023). This process should only begin once culturally relevant items and measures have been identified or adapted for the population of interest (Kagawa-Singer et al., 2015). When culturally relevant measures are not available, qualitative methods are useful for identifying relevant constructs (Kagawa-Singer et al., 2015).

Finally, there is a critical need to address existing sleep health disparities, which can only be done if both language and culture are considered in assessment tools (Jackson et al., 2020). To advance the field of sleep research around the world, and promote inclusivity in sleep research, investigators need to utilize best practices for translations and cultural adaptations.

## 2 Our process

In this paper, we present our recent experience applying best practices in a study with both Spanish and English-speaking Mexican American mothers of toddlers to highlight the value of this process. This work is part of a larger project measuring parental sleep-related beliefs and parenting practices in Mexican American parents of toddlers. To ensure the cultural and contextual relevance of survey measures for the larger study and to assure relevance to Mexican American parents of toddlers, we first conducted 20 individual semi-structured interviews and four focus groups (*n* = 23) with Spanish and English-speaking Mexican American parents exploring parental sleep-related beliefs and parenting practices. We used these qualitative findings to adapt existing survey-based measures as needed, create measures for newly identified constructs, and identify existing items not requiring revision. At the start of our translation process, we were thus working with sleep-related survey measures in three different stages of the translation and adaptation process: (1) newly developed or adapted measures, (2) existing measures without available translations, and (3) existing measures with available translations.

To conduct our work, we utilized a team-based approach to translation. We assembled a diverse, bilingual and bicultural team including individuals with expertise in pediatric sleep assessment and measurement development, disparities research, clinical pediatric practice, social psychology, cognitive interviewing, measurement development in Latino populations, and data collection in Latino communities. Almost all team members were bilingual, including native Spanish and native English speakers. A few team members had lived experience in Mexico and experience working with the local Mexican American community.

**Figure 1:**
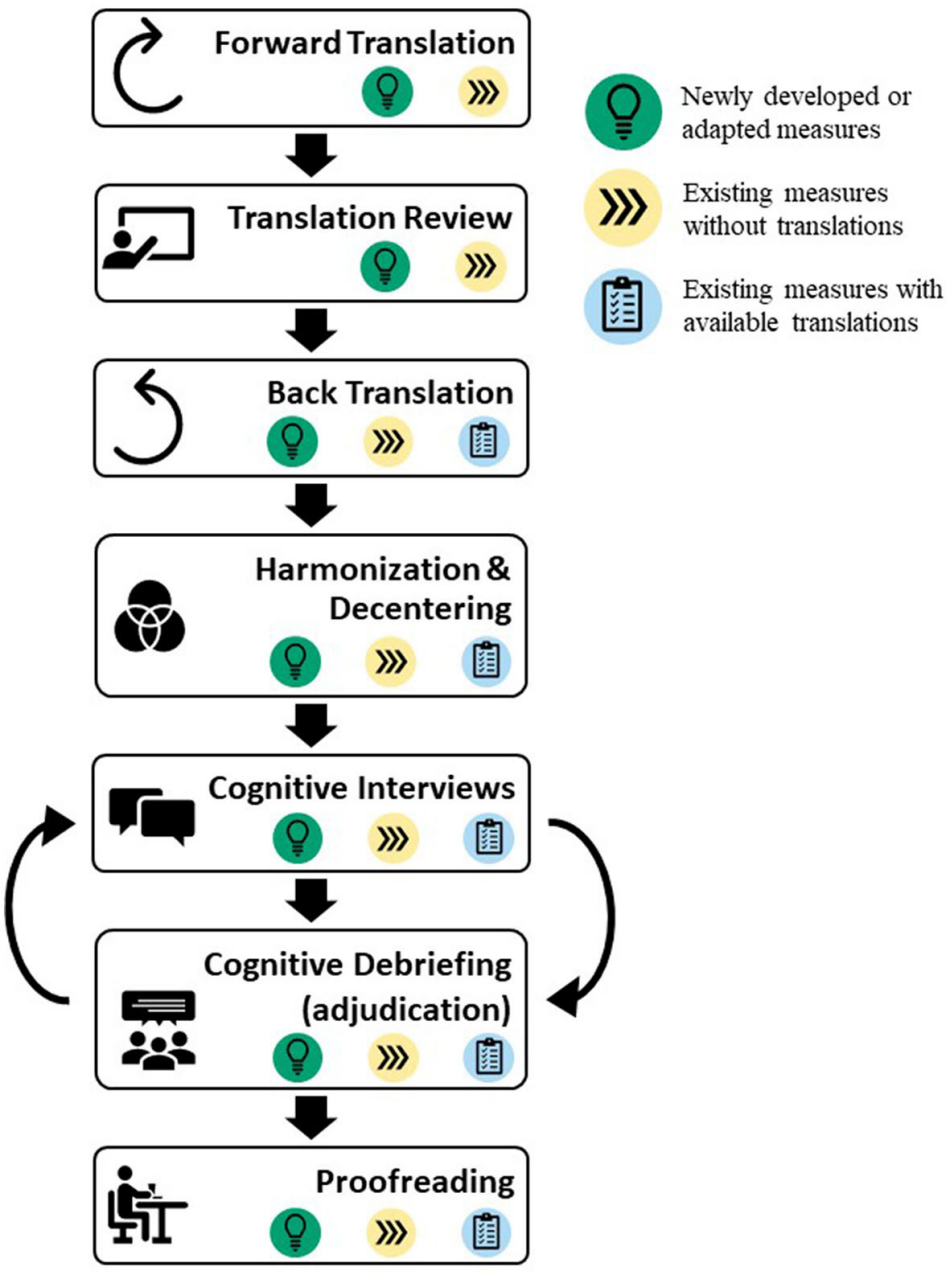
The multi-step process used to translate and adapt sleep-related survey items and questionnaires. Icons indicate which steps were used in translation and cultural adaptation of newly developed or adapted measures, existing measures without translations, and existing measures with available translations.

The steps we completed are shown in Figure 1. While newly developed or adapted measures, as well as existing measures without translations, were appraised in each step, existing measures with translations were only appraised in specific steps, as outlined in Figure 1. Below we present our methods, rationale, and findings for each step of the process. Examples of issues we identified and how we handled these are also presented in Table 1.

**Table 1:** Examples of cultural and linguistic adaptations made to sleep item translations during different stages of the translation process.

	**Item reviewed (English)**	**Item reviewed (Spanish)**	**Description of findings**	**Change(s) made**
**Forward translation, translation review, and back translation**				
1	It makes the parent sleep poorly	Hace que el padre duerma pobre	“Pobre” used to describe socioeconomic status or when expressing sympathy, rarely used when communicating quality of sleep	The adjective “pobre” replaced with “mal,” a more culturally relevant word used when describing sleep
2	In which room does your child usually fall asleep at bedtime? *In his or her own room/In parents' room/In sibling's or other bedroom/In another room of the house*	¿En qué habitación suele dormirse su hijo por la noche? *En su dormitorio/En el dormitorio de sus padres/En el dormitorio de un hermano u otro dormitorio/En otra habitación de la casa*	Two Spanish words (“dormitorio” and “habitación”) that translate to “bedroom” were used to express more general English word “room”	Spanish nouns “dormitorio” and “habitación” replaced with “cuarto” a more general noun used to describe any kind of room, not just a bedroom, in both question and response options
**Harmonization and decentering**				
3	It allows the parent to help the child in case anything happens	Le permite al papá a ayudarle a el/la niño/a en caso de que algo pase	“Papá” understood as “father,” does not reflect English word “parent” referring to either mother or father	The noun “al papá” changed to “el padre” a more accurate translation of parent. Because “padre” could still be understood to solely refer to fathers, we changed this to the plural “padres” in Spanish and “parents” in English to be inclusive of either mother or father
	**Item tested (English)**	**Item tested (Spanish)**	**Description of findings**	**Change(s) made**
**Cognitive interviewing, debriefing, adjudication, and proofreading**				
4	It makes the parents anxious that the child might need something	Le da ansiedad a los padres que el/la niño/a pueda necesitar algo	A participant suggested the word “ansiedad” may be unclear to some. Upon discussion, the team reflected on the social and cultural stigma of mental health within the Hispanic/Latino community and how anxiety is most often spoken of in a severe and negative context	The adjective “anxious” replaced with “worry” in English and the corresponding Spanish translation changed to “preocupar”
5	It makes the child feel bad	Hace que el/la niño/a se sienta mal	“Mal” was understood as a physical pain, discomfort, or that the child doesn't feel well	The adjective “bad/mal” was replaced with “sad/triste” to reflect the investigators intention of an emotional feeling of bad in English and Spanish
6	Does [child's name] snore during sleep? *Never (or only when sick or have a cold)/Only occasionally/Less than 3 times a week/3 times a week or more*	? [Child's name] ronca mientras duerme? *Nunca (o solo cuando está enfermo/a)/Sólo ocasionalmente (A veces)/Menos de 3 veces a la semana/3 veces a la semana o más*	Participants had difficultly selecting a response category because they did not understand how to interpret “only occasionally” and whether it was more than or less than the next category “less than 3 times a week”	Response options 2 & 3 were combined in both language versions, ensuring all response options were quantifiable and harmonizing across languages Only occasionally (less than 3 times a week)

### 2.1 Forward translation, translation review, and back translation

#### 2.1.1 Process

We forward translated items without existing translations from English into Spanish utilizing a professional service. A few items identified after we used this service were forward translated by a native Spanish-speaking bilingual team member with lived experience in Mexico and working experience with the local Mexican American community. This same team member reviewed the professional translations and existing translations, making minor revisions as needed to ensure that translations had the intended meaning and word choice for the local community. Another native Spanish-speaking bilingual and bicultural team member who was not familiar with the English or Spanish language versions then back translated new and adapted items into English. After this, a bilingual team member looked for discrepancies between the back-translated version and the original English version.

#### 2.1.2 Rationale

The use of a professional translator ensures that the forward translation is developed by someone skilled in creating translations. However, relying on one person to translate survey items, without additional review, is limited by the individual translator's own interpretation of items and possibly their preference for words not used in the population (Harkness, 2003). A team-based approach to translation is recommended to avoid these limitations (Harkness, 2003). Investigators use back translation as a way of evaluating whether the original and translated versions of survey items are comparable. This valuable step is a commonly used approach, with one study suggesting that over half of cross-cultural studies rely on forward and back translation alone (Cheng and Im, 2021). However, the translation process is not as simple as translating word-for-word from one language to the other. Translations must ensure that items are conceptually equivalent, linguistically accessible for the study sample, and culturally appropriate (Weech-Maldonado et al., 2001; Harkness et al., 2010; Schwarz et al., 2010; Cheng and Im, 2021).

#### 2.1.3 Findings

Upon review of the items that were professionally translated or had existing translations, only a handful of items were identified as needing changes. Our review also included ensuring that translations used the formal pronoun of “usted” for the word “you,” rather than the informal “tú.” The use of these pronouns varies across Spanish-speaking countries. In Mexican and Mexican American communities, the formal “usted” is the more commonly used pronoun and is used as a sign of respect when speaking with people one does not know well, which applies to research participants in our case. Two examples of additional changes made are presented below.

(a) The professional translation used the term “pobre” for the adverb “poorly” in the following item—“It makes the parent sleep poorly.” Since “pobre” is not a commonly used adjective to describe “poor sleep,” but instead used in the context of socioeconomic status or when expressing sympathy, we replaced the word “pobre” with “mal,” the more common word used to describe quality of sleep.
(b) An existing translation of the BISQ-R used two different nouns, “dormitorio” and “habitación,” for the word “room” in multiple items about sleeping arrangement (Mindell et al., 2019). These Spanish words refer specifically to a bedroom, while the intended meaning of the original English item is inclusive of any room. Both words were replaced with “cuarto,” the broader translation of the word “room,” to more accurately reflect the item's intended meaning *[Additionally, the word “dormitorio” is not commonly used within the Mexican and Mexican American community]*.

### 2.2 Harmonization and decentering

#### 2.2.1 Process

Our team member identified one discrepancy between the back-translated version and the original English version. This was subsequently verified through team review of the two versions assessed side by side. Of note, we approached this process using a decentering approach in which either the English or Spanish language versions could be adjusted to ensure cultural and linguistic equivalence across languages.

#### 2.2.2 Rationale

Harmonization encompasses the process of addressing translation discrepancies across language versions. By harmonizing across languages, investigators can combine data collected across languages (Wild et al., 2005). The decentering approach places equal value on both language versions of the instrument, revising the original or translated versions as needed to support conceptual equivalence and cultural relevance in both languages. This approach assumes that neither language version of the instrument is considered final until the end of the translation and adaptation process (Marin and Marin, 1991, p. 93). Decentering has been a recommended approach to cross-cultural research for decades (Marin and Marin, 1991; Harkness et al., 2010).

#### 2.2.3 Findings

The only discrepancy identified with back translation was with the word “parent” in English, which had been professionally translated into Spanish as “papá,” a term that is interpreted to mean father. To ensure that participants understood that we meant mother or father or both, we converted the items to the plural, using the term “parents” in English, “padres” in Spanish.

### 2.3 Cognitive interviews, debriefing, adjudication, and proofreading

#### 2.3.1 Process

Survey items were pretested in 42 cognitive interviews with 22 unique individuals. Interviews were conducted by phone by trained staff and lasted about 45 min each. Individuals who completed more than one interview were administered different sections of the interview each time. Following an informed consent process, staff introduced each participant to the interview format and purpose, and then reassured participants that there were no right or wrong answers. In each interview, interviewers read each survey item and then elicited feedback from the participant using a structured format with standardized questions and probes. To evaluate comprehension, participants were asked to repeat the item in their own words or, if needed, to share what they were thinking about when they heard the item. Participants were also asked if there was anything confusing about the item and after responding, whether it was easy or hard to answer. Staff used follow-up spontaneous non-standardized probes as needed to clarify participant responses (Lam and Valve, 2021, p. 170). Interviews were audio recorded and staff took notes using a structured interview template. Following each interview, staff identified items with comprehension or response concerns. Interviews were conducted in batches of 3–5 in each language followed by team debriefing meetings.

During weekly debriefing, the team used a collaborative analysis approach when reviewing the results of the cognitive interviews for each individual item (Willis, 2015). Any difficulties in comprehension or interpretation by a participant were discussed and the team adjudicated on the need for revisions, the changes to be made, or the addition of probes to the interview guide to further explore identified difficulties. We continued to apply a decentering approach throughout this process. Therefore, following any change made in one language, the team re-examined the same item in the other language to determine whether changes were needed to ensure linguistic equivalency. The linguistic equivalence of each item, original and revised, was designated as verified when tested with at least five different participants in each language without concerns in comprehension or interpretation. All revisions and adaptations were documented in a “modifications diary” detailing the changes and reasoning (Rabin et al., 2014, p. 75). Finally, after all items were validated, the team did a final proofreading of each measure to identify any unidentified grammar or spelling mistakes.

#### 2.3.2 Rationale

Cognitive interviews are a useful tool for ensuring conceptual equivalence and cultural relevance of survey items (Willis, 2005, p. 110; Kagawa-Singer et al., 2015; Lam and Valve, 2021). Through such interviews, investigators evaluate how potential participants “understand, mentally process and respond” to specific survey items, revising items in response and then retesting them in an iterative process (Willis, 2005, p. 3; Lam and Valve, 2021, p. 166). Cognitive pretesting is fundamental to identifying and addressing problems in item content and cultural relevance, response options, and questionnaire format that could influence a participant's response (Pan et al., 2010; Schwarz et al., 2010; Willis, 2015; Pennell and Cibelli Hibben, 2016; Vujcich et al., 2021). Across research, this method is underutilized, introducing the potential for error in participant interpretation and response to survey items (Willis, 2005; Castillo-Díaz and Padilla, 2013; Lyons-Thomas et al., 2014; Pennell and Cibelli Hibben, 2016; Lam and Valve, 2021, p. 166).

In cognitive debriefing, a collaborative approach safeguards against individual analyst bias that can result from separate and uncoordinated data interpretation. The collaborative approach results in high-quality translations and opportunities for cultural adaptation (Willis, 2015; Walde and Völlm, 2023). A diverse team with complimentary areas of expertise, such as our team, provides a wide range of perspectives when interpreting cognitive interview results and making determinations, an approach that results in culturally appropriate and clear items that maintain their originally intended meanings (Harkness and Schoua-Glusberg, 1998; Wild et al., 2005; Harkness et al., 2010; Willis, 2015; Vujcich et al., 2021; Judit et al., 2022).

#### 2.3.3 Findings

Below are few examples of item-level problems related to cultural concepts and translation that arose during cognitive interviews and the solutions we identified during debriefing. Item-level and questionnaire design issues not related to the translation process were also identified but are not presented here.

(a) We identified issues with the term “anxious,” which was used in an item to assess parent worry about the child. In the United States, for many people, the word “anxious” is part of everyday vernacular, used to express general feelings of concern. Meanwhile, in Latin American and Hispanic cultures where mental health is often stigmatized, the word “anxious” or “ansiedad” is rarely used. It most commonly refers to severe anxiety and carries negative connotations (Eghaneyan and Murphy, 2020). After one participant noted that the word might be unclear to some individuals, Latina team members initiated a conversation about this cultural difference and the use of the word “anxious” and “ansiedad.” The team agreed that the intent was to inquire about a general sense of concern; accordingly, the word “anxious” was replaced with “worry,” and in Spanish “ansiedad” was replaced with “preocupar.”
(b) For the item “It makes the child feel bad,” Spanish participants interpreted the direct professional translation, “Hace que el/la niño/a sienta mal” as a physical feeling of pain and discomfort or the child not feeling well. Given that the intended meaning was an emotional feeling of bad, the English and Spanish item was changed to “It makes the child feel sad” and “Hace que el/la niño/a sienta triste.”
(c) When asked the BISQ-R question “Does your child snore during sleep?/Never (or only when sick or have a cold), only occasionally, less than three times a week, three times a week or more,” multiple Spanish-speaking participants expressed difficulty selecting a response option. When probed they explained that they did not understand the difference between the option “only occasionally” and “less than three times a week,” and asked which option indicated a higher frequency in snoring. After team debriefing and the consultation of an expert in early childhood sleep, the two response options were combined in both Spanish and English versions to “only occasionally (less than three times a week),” ensuring that all response options were quantified and providing harmonization across languages. Following additional cognitive testing of the adapted item, participants did not have any difficulty answering the question or express any confusion regarding the response options.

## 3 Discussion

This paper highlights issues that need to be considered when translating new and existing sleep-related survey items. Our work underscores the importance of using a multi-step mixed methods approach including cognitive interviews with the target population to ensure that measures are conceptually equivalent across languages and culturally appropriate. With the increased appreciation of cultural differences in pediatric sleep practices, the growing focus on addressing existing sleep disparities, and the limited number of translated sleep measures, investigators conducting research across languages need to be prepared to implement a similar approach.

Had we only done forward and back translation, we would have been limited in ensuring conceptual equivalence across languages or cultural appropriateness of items. The additional steps taken after forward and back translation identified multiple item-level issues requiring revision that would have otherwise not been identified. Additional lessons from cognitive interviews and the cognitive debriefing process underscore the importance of an iterative process administering items, debriefing, revising the items, then readministering the revised items to ensure a complete evaluation of all changes. Moreover, a team-based approach was vital throughout our process, including having bilingual and bicultural staff members who administered the cognitive interviews participate in cognitive debriefing. Their direct and recent experience administering the items to the target population was valuable to team decision making. We advise researchers to implement these best practice methods. Minor adjustments to the process we present are possible if needed to enhance feasibility (e.g, use of a professional service to conduct back translations or conducting cognitive interviews face-to-face).

With any translation, the socio-cultural context of the target community must be considered (Akbari, 2013; Walde and Völlm, 2023). Linguistic cultural differences are influenced by behavioral and communication norms, education and literacy levels, and social norms and values (Akbari, 2013; Pennell and Cibelli Hibben, 2016). We started our consideration of the socio-cultural context with qualitative interviews and focus groups conducted prior to the translation process. Additionally, knowing that measures utilized in one population may not maintain the same relevance or meaning when administered in another population, despite being in the *same* language, we opted to appraise all items in cognitive interviews, including those that had been translated for previous studies. Using this approach, we identified several translation problems. We highly recommend this approach.

While the work we present is in line with best practices, we recognize the process is resource intensive. In addition to the time required to support such work, teams must have the required expertise in applying this approach, including content and methods expertise, native language skills in the relevant languages, and staff skilled in engaging with participants during the cognitive interview process. Investigators must consider these resource requirements in their project planning as they incorporate these steps into the study design. Moreover, it is essential for those who fund sleep and health disparities research to understand the resources required and to approve funding for translations and cultural adaptation that adhere to best practices guidelines, including starting with qualitative work when needed. Funding must be approved in spite of the increased costs associated with the development of linguistically and culturally appropriate measures compared to similar work conducted only in English.

## 4 Implications

Translations are required to ensure the advancement of the sleep field, promoting inclusivity and a broader geographic impact, as well as comparability of results across samples completing surveys in different languages. To ensure scientific rigor within the field of sleep research, investigators must utilize best practices for translations and cultural adaptations. Skipping such a process runs the risk of introducing errors in participant understanding or response through poorly done translations, possibly undermining the validity of current and future sleep research. These risks apply to both interventional and observational sleep research, both of which may include patient- or parent-reported measures. Forward and back translation is not enough and should be considered inadequate within the field of sleep research.

## Data availability statement

The datasets presented in this article are not readily available because a data sharing agreement is required. Requests to access the datasets should be directed to DT, Darcy.Thompson@cuanschutz.edu.

## Ethics statement

The studies involving humans were approved by Colorado Multiple Institutional Review Board. The studies were conducted in accordance with the local legislation and institutional requirements. The Ethics Committee/institutional review board waived the requirement of written informed consent for participation from the participants or the participants' legal guardians/next of kin because the research presents no more than minimal risk of harm to subjects and involves no procedures for which written consent is required outside of the research context.

## Author contributions

DT: Conceptualization, Formal analysis, Funding acquisition, Investigation, Methodology, Project administration, Supervision, Writing – original draft, Writing – review & editing. MF: Formal analysis, Investigation, Project administration, Visualization, Writing – original draft, Writing – review & editing. EM: Formal analysis, Investigation, Project administration, Visualization, Writing – original draft, Writing – review & editing. JT: Conceptualization, Formal analysis, Investigation, Writing – review & editing, Methodology. LM: Conceptualization, Formal analysis, Funding acquisition, Investigation, Methodology, Supervision, Writing – original draft, Writing – review & editing.
